# Clinical and economic burden associated with graft-versus-host disease following allogeneic hematopoietic cell transplantation in France

**DOI:** 10.1038/s41409-023-01930-8

**Published:** 2023-02-10

**Authors:** David Michonneau, Nadia Quignot, Heng Jiang, Dawn Reichenbach, Maebh Kelly, Anita Burrell, Xiang Zhang, Kris Thiruvillakkat, Mohamad Mohty

**Affiliations:** 1 Hospital Saint-Louis, Hematology and Transplantation Unit, Paris, France; 2 Certara France, Evidence & Access, 54 Rue de Londres, 75008 Paris, France; 3 CSL Behring, King of Prussia, PA USA; 4 CSL Behring, West Sussex, UK; 5 Anita Burrell Consulting, Flemington, NJ 08822-1907 USA; 6 Hospital Saint Antoine, Sorbonne University and INSERM UMRs 938, Paris, France

**Keywords:** Stem-cell therapies, Graft-versus-host disease

## Abstract

The real-world clinical and economic burden of graft-versus-host disease (GVHD) following allogeneic hematopoietic stem cell transplantation has not been comprehensively studied in France. Clinical outcomes, healthcare resource utilization and costs associated with acute GVHD (aGVHD), chronic GVHD (cGVHD), acute plus chronic GVHD (a+cGVHD) versus no GVHD were compared using French administrative claims data. After propensity score matching, 1934, 408, and 1268 matched pairs were retained for the aGVHD, cGVHD, and a+cGVHD cohorts, respectively. Compared with patients with no GVHD, odds of developing severe infection were greater in patients with aGVHD (odds ratio: 1.7 [95% confidence interval: 1.4, 2.1]). Compared with patients with no GVHD, mortality rates were higher in patients with aGVHD (rate ratio (RR): 1.6 [1.4, 1.7]) and with a+cGVHD (RR: 1.1 [1.0, 1.2]) but similar in patients with cGVHD (RR: 0.9 [0.7, 1.1]). Mean overnight hospital admission rates per patient-year were significantly higher in patients with aGVHD and a+cGVHD compared with no GVHD. Total direct costs (range €174,482–332,557) were 1.2, 1.5, and 1.9 times higher for patients with aGVHD, cGVHD, and a+cGVHD, respectively, versus patients with no GVHD. These results highlight the significant unmet need for effective treatments of patients who experience GVHD.

## Introduction

Hematopoietic stem cell transplantation (HSCT) is an effective and, in some cases, the only treatment option for many patients with hematological malignancies [1]. Advances in the field of allogeneic HSCT (allo-HSCT), in which patients receive stem cells from an unrelated donor, have greatly increased the number of transplants performed over the past 3 decades, and now ~20,000 procedures are performed annually across Europe, including more than 2000 in France [2, 3]. Despite such advances, graft-versus-host disease (GVHD) remains the most frequent and potentially fatal complication, occurring in ~40% of allo-HSCT recipients [4].

GVHD is traditionally categorized as either acute (aGVHD), usually presenting within 100 days of transplantation, or chronic (cGVHD), which more frequently occurs 100 days after transplantation [5]. Both aGVHD and cGVHD carry substantial health and economic burdens. Whereas cGVHD is associated with long-term morbidity and mortality, aGVHD is the primary fatal complication within the first few months following allo-HSCT [6]. Compared with patients who do not develop GVHD after allo-HSCT, those with GVHD have been shown to have higher hospital readmission, infection, and associated mortality rates [7, 8]. The clinical implications of GVHD have been shown to translate to an increased economic burden in the United States (US) with higher readmission rates for patients with GVHD compared with no GVHD, and a longer median length of hospital stay as well as higher median total costs for the initial procedure [8–11].

An unmet need exists for effective therapies to prevent and treat GVHD following allo-HSCT [12]. Treatment options for GVHD are largely limited to systemic corticosteroids and, specifically for patients with cGVHD, immunosuppressants [13]. Currently, there is no standard second-line treatment for patients who become resistant to or dependent on corticosteroids. A full evaluation of the public health burden of patients with GVHD is an important component for understanding the disease and its management. To date, the clinical and economic burden of GVHD has not been comprehensively studied in France. Quantifying the risk of severe disease, mortality, and the economic burden of GVHD is useful for healthcare providers, regulators, and payers. We performed a real-world analysis of the clinical outcomes, healthcare resource utilization (HCRU), and costs associated with aGVHD, cGVHD, and acute plus chronic (a+cGVHD) GVHD in France.

## Materials and methods

### Data source and study design

This study was a retrospective, nationwide cohort study using administrative claims obtained from the French national health data system, Système National des Données de Santé (SNDS). The SNDS contains health records of an estimated 65 million insured individuals. The French national health system offers universal coverage, so this is a representative sample of the whole population. SNDS data are linked via unique identifiers to primary care, hospital, pharmacy, and death registration databases, allowing for the linkage of patient treatment history, treatment patterns, and hospitalization based on International Classification of Diseases, 10th revision (ICD-10) codes.

The study period ran from January 1, 2011 through December 31, 2019. The index date was defined as the date of the first allo-HSCT procedure between January 1, 2012 and December 31, 2018. The baseline period was defined as the 12 months prior to the index date (as early as January 1, 2011). The follow-up period was a minimum of 12 months after the index date, until the last available information, death, or the end of the study (December 31, 2019), whichever came first.

This study was conducted in accordance with legal and regulatory requirements, as well as with scientific purpose, value, and rigor. The study followed generally accepted research practices described in the Guidelines for Good Pharmacoepidemiology Practices issued by the International Society for Pharmacoepidemiology, Good Epidemiological Practice guidelines issued by the International Epidemiological Association, Good Practices for Outcomes Research issued by the International Society for Pharmacoeconomics and Outcomes Research, International Ethical Guidelines for Epidemiological Research issued by the Council for International Organizations of Medical Sciences, and the European Medicines Agency, as well as the European Network of Centres for Pharmacoepidemiology, and Pharmacovigilance Guide on Methodological Standards in Pharmacoepidemiology. The final protocol was reviewed and approved by a scientific committee and the national data protection agency. All patient data were pseudonymized, which according to applicable legal requirements renders the data exempt from privacy laws; therefore, obtaining informed consent from patients was not required.

### Study population

Patients (aged 18 years and above at the index date) who underwent allo-HSCT for any hematological malignancy between January 1, 2012 and December 31, 2018 were eligible for the study. Patients were excluded if they had an allo-HSCT prior to the start of the study or multiple allo-HSCT procedures during the study period. All patients were required to have at least 12 months of baseline data prior to the allo-HSCT and 12 months of possible follow-up data (unless they died). Eligible patients were identified in the database using the common classification of medical procedure codes or, where applicable, diagnosis-related group or ICD-10 codes.

Using ICD-10 codes, patients were divided into 1 of 4 categories by GVHD type: aGVHD, cGVHD, a+cGVHD, or no GVHD (Supplementary Table 1).

### Outcome measures

The outcomes measures assessed were rates of severe infection, mortality, HCRU, and healthcare costs. Severe infections were defined as those leading to hospitalization and were identified through ICD-10 discharge codes. Relapse—investigated as an exploratory outcome—was defined as any hospital readmission for the same underlying malignancy, followed by cancer treatment. Mortality was defined as all-cause death.

### Statistical analysis

Continuous variables were summarized descriptively with mean, standard deviation (SD), median, minimum and maximum, and lower and upper quartiles (Q1; Q3). Frequencies and percentages were reported for categorical variables. The chi-square test was used for categorical variables.

The crude mortality rate was calculated as a ratio of the number of deaths during the follow-up period divided by the total person-years in the given cohort. The crude rates of HCRU and of severe infections were calculated as the total number of events divided by the person-years. A mean value was then estimated for each cohort.

No imputed data were used for missing values for outcomes assessments. Statistical analyses were conducted using SAS (version 9.4 TS1M4). Percentages were based on available observations (known values), and outliers were included in ranges and percentiles. The rate ratios (RRs) were calculated using the OpenEpi statistical tool [14].

### Comparative analysis

For the comparative analysis, separate 1:1 propensity score matching was used to balance covariates between the aGVHD, cGVHD, and a+cGVHD groups versus the no GVHD group. The propensity score, defined as the probability of a patient to develop GVHD conditional on observed baseline covariates, was estimated using a logistic regression model. The baseline covariates explored were hematological condition for the allo-HSCT, age, gender, comorbidities, and preparative regimens. The final model was chosen based on the Akaike information criterion (a mathematical method for evaluating how well a model fits the data it was generated from) and the sample size retained for each comparison. The final covariates were selected based on clinical relevance and/or statistical significance.

### Binary and continuous demographics

All binary outcomes were described using frequencies. The excess number of infections (viral, fungal, bacterial, or other infection), the excess number of deaths, and the excess number of HCRUs were assessed using conditional logistic regression stratified by the paired identifier. Odds ratios (ORs) with the associated 95% confidence intervals (CIs) and two-sided *p* values were estimated.

### Time-to-event outcomes

The effect of GVHD on each time-to-event outcome of interest (time to first severe infection and time to death) was summarized using Kaplan–Meier (KM) methodology on the matched population. The assumption of proportional hazards was evaluated by visually inspecting the KM plot and confirmed by testing the significance of interactions between treatment and the log of time. Hazard ratios (HRs) were used to assess excess time to death (or end of study follow-up, whichever came first), and excess time to severe infections.

### Cost outcomes

Actual costs reimbursed were considered (without inflation to a standard cost year). Cost data were not normally distributed. The effect of GVHD on costs was investigated in the propensity score-matched populations using the generalized linear models with gamma distribution and log-link function. Excess cost was assessed using mean differences and cost ratios, together with the associated 95% CIs and *p* values.

## Results

**Fig. 1:**
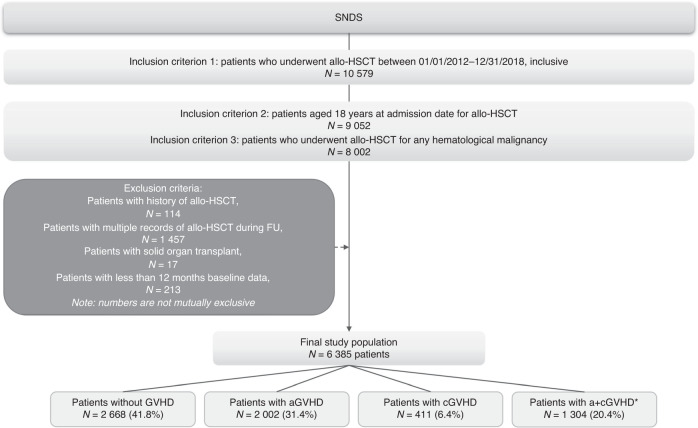
Study flow chart. aGVHD acute GVHD, a+cGVHD acute and chronic GVHD, allo-HSCT allogeneic hematopoietic stem cell transplantation, cGVHD chronic GVHD, FU follow-up, GVHD graft-versus-host disease, ICD-10 International Classification of Diseases, 10th revision, SNDS Système National des Données de Santé. *Includes patients with both an episode of acute and chronic GVHD at some point during follow-up. Identified using ICD-10 codes.

A total of 10,579 patients were identified in the SNDS database as recipients of allo-HSCT during the study period. After applying inclusion and exclusion criteria, 6385 patients were included in the study population (Fig. 1). The mean age of the overall study population was 51.1 years and 57.9% were male. A total of 2002 patients (31.4%) experienced aGVHD, 411 patients (6.4%) had cGVHD, and 1304 patients (20.4%) had a+cGVHD. The remaining 2668 patients (41.8%) had no recorded diagnosis code for GVHD (Supplementary Table 2).

Patients with GVHD had lower rates of relapse than those with no GVHD. Overall, 16.3% of the total study population (1043 patients) had a relapse (aGVHD 276 patients [13.7%]; cGVHD 61 patients [14.8%]; a+cGVHD 220 patients [16.8%]; no GVHD 486 patients [18.2%]). Among all patients who had a relapse, the median time to relapse was 1.2 (range 0.1–56.5) months.

The final covariates selected to pair for propensity score matching for all groups were age at allo-HSCT, gender, year of allo-HSCT, and preparative regimen (use of clofarabine). Additional covariates for each GVHD type included related donor, other preparative regimen (use of carmustine), and the presence of diabetes for aGVHD and a+cGVHD; lymphoid leukemia, acute myeloblastic leukemia, and total body irradiation for aGVHD; congestive heart failure and connective tissue disorder for cGVHD; and cerebrovascular disease and any tumor (including lymphoma and leukemia except for malignant neoplasm of the skin) for a+cGVHD.

After propensity score matching, 1934 matched pairs were retained for the aGVHD cohort; 408 matched pairs were retained for the cGVHD cohort and 1268 matched pairs were retained for the a+cGVHD cohort (Table 1). The median ages of each of the matched cohorts versus no GVHD were 55.0 (range 18.0–77.0) for the aGVHD cohort (54.0 [range 18.0–78.0] no GVHD); 54.0 (range 18.0–75.0) for the cGVHD cohort (52.5 [range 18.0–78.0] no GVHD); and 53.0 (range 18.0–76.0) for the a+cGVHD cohort (54.0 [range 18.0–78.0] no GVHD).

**Table 1:** Patient characteristics after propensity score matching.

	aGVHD (*N* = 1934)	No GVHD (*N* = 1934)	Std diff^a^	cGVHD (*N* = 408)	No GVHD (*N* = 408)	Std diff	a+cGVHD (*N* = 1268)	No GVHD (*N* = 2268)	Std diff
Hematologic condition for allo-HSCT^b^ (*n*, %)									
Myeloid leukemia	878 (45.4)	872 (45.1)	0.006	200 (49.0)	213 (52.2)	−0.064	603 (47.6)	1335 (50.0)	−0.019
Acute myeloblastic leukemia	745 (38.5)	749 (38.7)	−0.004	176 (43.1)	182 (44.6)	−0.030	504 (39.8)	1175 (44.0)	−0.061
Lymphoid leukemia	377 (19.5)	372 (19.2)	0.007	68 (16.7)	74 (18.1)	−0.039	222 (17.5)	434 (16.3)	0.025
Myelodysplastic syndromes	296 (15.3)	253 (13.1)	0.064	56 (13.7)	45 (11.0)	0.082	177 (14.0)	333 (12.5)	0.005
Acute lymphoblastic leukemia	285 (14.7)	281 (14.5)	0.006	41 (10.0)	55 (13.5)	−0.107	148 (11.7)	321 (12.0)	−0.024
Non-Hodgkin lymphoma	104 (5.4)	137 (7.1)	−0.071	23 (5.6)	21 (5.2)	0.022	68 (5.4)	169 (6.3)	−0.027
Multiple myeloma and plasma cell neoplasms	95 (4.9)	118 (6.1)	−0.052	24 (5.9)	26 (6.4)	−0.020	69 (5.4)	157 (5.9)	−0.024
Hodgkin lymphoma	88 (4.6)	83 (4.3)	0.013	18 (4.4)	18 (4.4)	0	63 (5.0)	115 (4.3)	0.004
Monocytic leukemia	60 (3.1)	58 (3.0)	0.006	11 (2.7)	11 (2.7)	0	43 (3.4)	75 (2.8)	0.027
Chronic lymphocytic leukemia	53 (2.7)	56 (2.9)	−0.009	16 (3.9)	9 (2.2)	0.100	48 (3.8)	69 (2.6)	0.082
Acute myelomonocytic leukemia	34 (1.8)	35 (1.8)	−0.004	13 (3.2)	7 (1.7)	0.095	27 (2.1)	48 (1.8)	0.060
Chronic myeloid leukemia	30 (1.6)	34 (1.8)	−0.016	Freq <5	12 (2.9)	−0.165	32 (2.5)	46 (1.7)	0.010
Not specified^c^	34 (1.8)	35 (1.8)	−0.004	8 (2.0)	Freq <5	0.200	17 (1.3)	42 (1.6)	0.014
Other and unspecified malignant neoplasms of the lymphatic, hematopoietic, and related tissues	Freq <5	6 (0.3)	−0.046	Freq <5	Freq <5	0	6 (0.5)	8 (0.3)	0.056
Year of allo-HSCT (*n*, %)									
2012	188 (9.7)	204 (10.6)	−0.027	57 (14.0)	63 (15.4)	−0.042	138 (10.9)	293 (11.0)	−0.008
2013	262 (13.6)	259 (13.4)	0.005	61 (15.0)	63 (15.4)	−0.014	180 (14.2)	366 (13.7)	−0.002
2014	283 (14.6)	287 (14.8)	−0.006	54 (13.2)	49 (12.0)	0.037	200 (15.8)	395 (14.8)	−0.013
2015	294 (15.2)	286 (14.8)	0.012	61 (15.0)	66 (16.2)	−0.034	190 (15.0)	400 (15.0)	−0.013
2016	289 (14.9)	292 (15.1)	−0.004	60 (14.7)	56 (13.7)	0.028	194 (15.3)	373 (14.0)	0.011
2017	289 (14.9)	285 (14.7)	0.006	52 (12.8)	56 (13.7)	−0.029	194 (15.3)	399 (15.0)	0.031
2018	329 (17.0)	321 (16.6)	0.011	63 (15.4)	55 (13.5)	0.056	172 (13.6)	442 (16.6)	−0.007
Age at allo-HSCT (*n*, %)									
Mean (SD)	51.1 (13.8)	50.9 (14.1)	0.016	51.5 (13.0)	49.7 (14.1)	0.129	50.8 (13.2)	51.1 (13.9)	0.004
Median (Q1, Q3)	55.0 (42.0, 62.0)	54.0 (41.0, 62.0)	–	54.0 (42.0, 62.0)	52.5 (40.0, 60.0)	–	53.0 (42.0, 62.0)	54.0 (42.0, 62.0)	–
Range (min, max)	(18.0, 77.0)	(18.0, 78.0)	–	(18.0, 75.0)	(18.0, 78.0)	–	(18.0, 76.0)	(18.0, 78.0)	–
Age group									
18 to <25 years	117 (6.0)	120 (6.2)	−0.007	13 (3.2)	21 (5.2)	−0.098	59 (4.6)	150 (5.6)	−0.060
25 to <45 years	424 (21.9)	455 (23.5)	−0.038	113 (27.7)	120 (29.4)	−0.038	308 (24.3)	615 (23.0)	−0.009
45 to <65 years	1062 (54.9)	1037 (53.6)	0.026	211 (51.7)	205 (50.2)	0.029	721 (56.9)	1455 (54.5)	0.108
≥65 years	331 (17.1)	322 (16.6)	0.012	71 (17.4)	62 (15.2)	0.060	180 (14.2)	448 (16.8)	−0.099
Age at first GVHD (*n*, %)									
Mean (SD)	51.2 (13.8)	–	–	52.2 (13.1)	–	–	51.0 (13.2)	–	–
Median (Q1, Q3)	55.0 (42.0, 62.0)	–	–	55.0 (43.0, 63.0)	–	–	54.0 (42.0, 62.0)	–	–
Range (min, max)	(18.0, 77.0)	–	–	(18.0, 75.0)	–	–	(18.0, 76.0)	–	–
Age group (*n*, %)		–	–		–	–		–	–
18 to <25 years	116 (6.0)	–	–	12 (2.9)	–	–	59 (4.7)	–	–
25 to <45 years	421 (21.8)	–	–	105 (25.7)	–	–	302 (23.8)	–	–
45 to <65 years	1064 (55.0)	–	–	205 (50.2)	–	–	717 (56.6)	–	–
≥65 years	333 (17.2)	–	–	86 (21.1)	–	–	190 (15.0)	–	–
Gender (*n*, %)									
Male	1122 (58.0)	1136 (58.7)	−0.015	217 (53.2)	205 (50.2)	0.059	736 (58.0)	1560 (58.5)	0.029
Female	812 (42.0)	798 (41.3)	–	191 (46.8)	203 (49.8)	–	532 (42.0)	1108 (41.5)	–
Donor typing (*n*, %)									
Donor: related	462 (24.0)	447 (23.1)	–	113 (27.7)	115 (28.2)	–	299 (23.6)	748 (28.0)	–
Donor: unrelated	737 (38.1)	697 (36.0)	–	140 (34.3)	153 (37.5)	–	440 (34.7)	875 (32.8)	–
Donor: NA	751 (38.8)	814 (42.1)	–	158 (38.7)	145 (35.5)	–	538 (42.4)	1073 (40.2)	–
Preparative regimens^d^ (*n*, %)									
Yes	1759 (91.0)	1760 (91.0)	−0.002	379 (93.0)	374 (91.7)	0.046	1137 (89.7)	2435 (91.3)	0.013
Busulfan	1374 (71.0)	1368 (70.7)	0.007	308 (75.5)	304 (74.5)	0.023	911 (71.8)	1944 (72.9)	0.018
Total body irradiation	384 (19.9)	391 (20.2)	−0.009	71 (17.4)	68 (16.7)	0.020	227 (17.9)	482 (18.1)	−0.002
Alemtuzumab	Freq <5	Freq <5	−0.046	Freq <5	Freq <5	−0.070	Freq <5	Freq <5	−0.040
Clofarabine	79 (4.1)	77 (4.0)	0.005	11 (2.7)	13 (3.2)	−0.029	34 (2.7)	153 (5.7)	0.005
Cytarabine	Freq <5	Freq <5	0	Freq <5	Freq <5	0.070	Freq <5	Freq <5	0
Not captured	175 (9.1)	174 (9.0)	–	29 (7.1)	34 (8.3)	–	131 (10.3)	233 (8.7)	–
GVHD prophylaxis^e^ (*n*, %)									
Yes	637 (32.9)	1572 (81.3)	−1.119	375 (91.9)	347 (85.0)	0.216	533 (42.0)	2164 (81.1)	−0.888
Ciclosporin	593 (30.7)	1499 (77.5)	−1.065	350 (85.8)	333 (81.6)	0.113	494 (39.0)	2064 (77.4)	−0.828
Mycophenolate mofetil	324 (16.8)	601 (31.1)	−0.341	141 (34.6)	118 (28.9)	0.121	242 (19.1)	845 (31.7)	−0.257
Tacrolimus	39 (2.0)	89 (4.6)	−0.145	53 (13.0)	19 (4.7)	0.297	43 (3.4)	123 (4.6)	−0.107
Sirolimus	5 (0.3)	12 (0.6)	−0.055	10 (2.4)	Freq <5	0.137	5 (0.4)	16 (0.6)	−0.051
Antithymocyte immunoglobulin	Freq <5	Freq <5	0	Freq <5	Freq <5	0	Freq <5	Freq <5	0
Methotrexate	Freq <5	Freq <5	0	5 (1.2)	Freq <5	0.158	Freq <5	Freq <5	0.040
Not captured	1 297 (67.1)	362 (18.7)	–	33 (8.1)	61 (15.0)	–	735 (58.0)	504 (19.0)	–
Comorbidities^f^ (*n*, %)									
Any tumor (including lymphoma and leukemia except for malignant neoplasm of skin)	1741 (90.0)	1784 (92.2)	−0.009	369 (90.4)	384 (94.1)	−0.046	1154 (91.0)	2475 (92.8)	−0.044
Diabetes	197 (10.2)	192 (9.9)	0.053	44 (10.8)	44 (10.8)	0.070	150 (11.8)	240 (9.0)	0.040
Chronic pulmonary disease	168 (8.7)	154 (8.0)	0.026	34 (8.3)	40 (9.8)	−0.051	106 (8.4)	220 (8.2)	0.012
Congestive heart failure	153 (7.9)	172 (9.0)	−0.035	22 (5.4)	23 (5.6)	−0.011	94 (7.4)	243 (9.1)	−0.066
Moderate to severe liver disease	92 (4.8)	89 (4.6)	0.007	12 (2.9)	16 (3.9)	−0.054	47 (3.7)	120 (4.5)	−0.020
Cerebrovascular disease	82 (4.2)	91 (4.7)	−0.023	14 (3.4)	18 (4.4)	−0.051	20 (1.6)	119 (4.5)	−0.006
Mild liver disease	62 (3.2)	62 (3.2)	0	18 (4.4)	11 (2.7)	0.093	43 (3.4)	78 (2.9)	0.013
Moderate or severe renal disease	59 (3.1)	62 (3.2)	−0.035	9 (2.2)	12 (3.0)	−0.089	30 (2.4)	77 (2.9)	−0.029
Metastatic solid tumor	57 (3.0)	63 (3.3)	0	15 (3.7)	10 (2.5)	0.070	45 (3.6)	84 (3.2)	−0.033
Peripheral vascular disease	34 (1.8)	37 (1.9)	−0.012	Freq <5	7 (1.7)	−0.064	22 (1.7)	49 (1.8)	0.006
Myocardial infarction	31 (1.6)	43 (2.2)	−0.045	6 (1.5)	7 (1.7)	−0.020	15 (1.2)	57 (2.1)	−0.040
Hemiplegia	30 (1.6)	39 (2.0)	0.009	Freq <5	7 (1.7)	0	13 (1.0)	52 (2.0)	0.005
Ulcer disease	28 (1.4)	17 (0.9)	0.053	6 (1.5)	Freq <5	0.070	12 (1.0)	22 (0.8)	0.008
Connective tissue disease	11 (0.6)	10 (0.5)	0.007	6 (1.5)	Freq <5	0.133	14 (1.1)	15 (0.6)	0.024
HIV/AIDS	5 (0.3)	Freq <5	−0.078	Freq <5	Freq <5	−0.138	Freq <5	Freq <5	0.006
Dementia	Freq <5	Freq <5	0.019	Freq <5	Freq <5	0	Freq <5	Freq <5	0.040
Diabetes with end-organ damage	Freq <5	Freq <5	−0.018	Freq <5	Freq <5	0.071	Freq <5	Freq <5	0.026
CCI (12 months prior to index date)									
Mean (SD)	2.6 (1.7)	2.7 (1.7)	−0.038	2.6 (1.7)	2.6 (1.5)	−0.028	2.6 (1.7)	2.7 (1.7)	−0.007
Median (Q1, Q3)	2.0 (2.0, 3.0)	2.0 (2.0, 3.0)	–	2.0 (2.0, 3.0)	2.0 (2.0, 3.0)	–	2.0 (2.0, 3.0)	2.0 (2.0, 3.0)	–
Range (min, max)	(0.0, 12.0)	(0.0, 15.0)	–	(0.0, 11.0)	(0.0, 11.0)	–	(0.0, 11.0)	(0.0, 15.0)	–
CCI category (*n*, %)									
0	126 (6.5)	101 (5.2)	0.055	29 (7.1)	16 (3.9)	0.140	82 (6.5)	136 (5.1)	−0.019
1	44 (2.3)	29 (1.5)	0.057	9 (2.2)	Freq <5	0.098	16 (1.3)	33 (1.2)	−0.014
2	1078 (55.7)	1103 (57.0)	−0.026	236 (57.8)	242 (59.3)	−0.030	752 (59.3)	1530 (57.4)	0.072
3	372 (19.2)	386 (20.0)	−0.018	78 (19.1)	91 (22.3)	−0.079	231 (18.2)	549 (20.6)	−0.064
≥4	314 (16.2)	315 (16.3)	−0.001	56 (13.7)	55 (13.5)	0.007	187 (14.8)	420 (15.7)	−0.011
Follow-up from first allo-HSCT (months)									
Mean (SD)	27.2 (26.9)	33.0 (27.6)	−0.218	41.1 (26.7)	38.0 (28.7)	0.111	35.7 (25.5)	32.7 (27.8)	0.025
Median (Q1, Q3)	16.1 (4.4, 46.2)	26.1 (7.5, 54.7)	–	36.8 (17.6, 64.1)	33.6 (11.6, 61.1)	–	30.1 (14.0, 53.9)	25.3 (7.1, 55.2)	–
Range (min, max)	(0.0, 94.8)	(0.0, 95.4)	–	(0.3, 95.1)	(0.1, 95.2)	–	(0.9, 95.4)	(0.0, 95.4)	–

*aGVHD* acute GVHD, *a+cGVHD* acute GVHD and chronic GVHD, *AIDS* acquired immunodeficiency syndrome, *allo-HSCT* allogeneic hematopoietic stem cell transplantation, *ANOVA* analysis of variance, *CCI* Charlson Comorbidity Index, *cGVHD* chronic GVHD, *Freq* frequency, *GVHD* graft-versus-host disease, *HIV* human immunodeficiency virus, *HSCT* hematopoietic stem cell transplantation, *max* maximum, *min* minimum, *NA* not applicable due to low sample size, *Q* quartile, *SD* standard deviation, *std diff* standardized difference.

^a^Std diff versus no GVHD. For continuous variables, if the normality test showed that the normal distribution assumption was not true, a Kruskal–Wallis test was used. If normality and homogeneity of variance were satisfied, then a one-way ANOVA test was used. A chi-square test was used for categorical variables. If 25% of the cells had expected counts of <5, then a Fisher exact test was applied.

^b^Most recent hematological condition before admission date of allo-HSCT (including date of allo-HSCT); conditions were exclusive.

^c^Any patient with different malignancy conditions on the same day was regarded as not specified.

^d^Preparative regimens should be recorded between initial hospital admission and actual date of allo-HSCT procedure (including the date of allo-HSCT); regimens were not exclusive. Cyclophosphamide, melphalan, carmustine, thiotepa, antithymocyte immunoglobulin, treosulfan, fludarabine, and etoposide were also looked at but were not presented as *n* < 5 for each, and the std diff was 0 across all groups.

^e^GVHD prophylaxis was administrated before the first documented GVHD and after the first allo-HSCT for patients with any type of GVHD; GVHD prophylaxis for the no GVHD group focused on prophylaxis within 100 days after the first allo-HSCT.

^f^At least one comorbidity within 1 year before the first allo-HSCT; these comorbidities were not mutually exclusive.

### Clinical outcomes

**Fig. 2:**
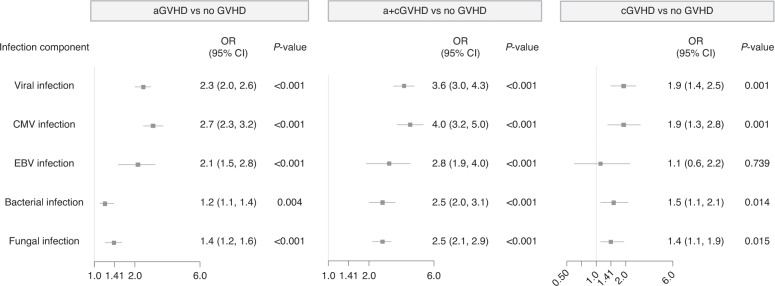
Odds ratios of severe infection, by GVHD type. aGVHD acute GVHD, a+cGVHD acute and chronic GVHD, cGVHD chronic GVHD, CI confidence interval, CMV *cytomegalovirus*, EBV Epstein-Barr virus, GVHD graft-versus-host disease, OR odds ratio.

Overall, patients with any GVHD type were more likely to develop infections than those with no GVHD (Fig. 2). Among patients with aGVHD, 88.9% (1720 patients) developed at least one severe infection, compared with 82.2% (1589 patients) in the no GVHD cohort (OR 1.7 [95% CI 1.4, 2.1], *p* < 0.001), and 30.6% (592 patients) had four or more severe infections, compared with 18.5% (357 patients) in the no GVHD cohort (OR 1.9 [95% CI 1.7, 2.3], *p* < 0.001). Although a numerically greater proportion of patients with cGVHD than no GVHD developed severe infection (85.3% versus 81.9%, respectively), the difference was not statistically significant (OR 1.3 [95% CI 0.9, 1.9], *p* = 0.179). Significantly more patients with cGVHD had four or more infections compared with no GVHD (33.6% versus 18.4%, respectively; OR 2.2 [95% CI 1.6, 3.0], *p* < 0.001). Significantly more patients with a+cGVHD had severe infection compared with no GVHD (94.2% versus 81.4%; OR 4.0 [95% CI 3.0, 5.4], *p* < 0.001), and significantly more patients with a+cGVHD had four or more infections compared with no GVHD (50.5% versus 20.0%, respectively; OR 4.0 [95% CI 3.3, 4.9], *p* < 0.001). The most common infections were bacterial, recorded in >70% of all patients; and viral infections recorded in 47.4%, 44.9%, 59.1%, and 27.5–31.9% of patients with aGVHD, cGVHD, a+cGVHD, and no GVHD, respectively. The most frequent viral infection was CMV, reported in 28.6%, 23.5%, 36% of patients with aGVHD, cGVHD, a+cGVHD respectively versus 12.5%–13.7% of patients with no GVHD (Supplementary Table 3).

Patients with aGVHD and a+cGVHD had an increased rate of hospitalization for severe infection, with an RR of 1.3 (95% CI 1.2, 1.4) and 1.1 (95% CI 1.1, 1.2), respectively, versus no GVHD. The rate of hospitalization for severe infection was similar for patients with cGVHD compared with no GVHD (RR 1.0 [95% CI 0.8, 1.1], *p* > 0.05).

**Fig. 3:**
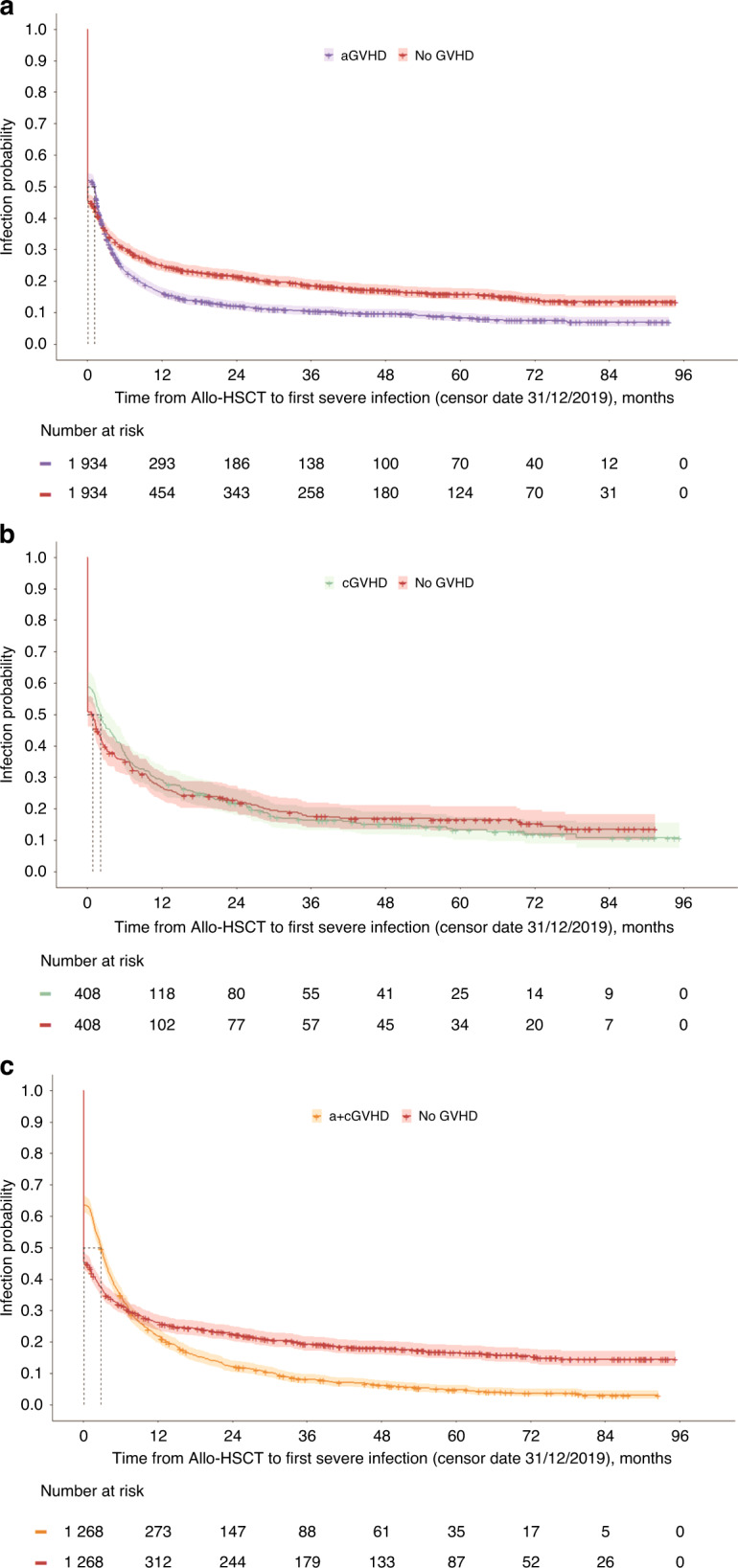
Time to first infection. **a** aGVHD versus No GVHD. **b** cGVHD versus No GVHD. **c** a + cGVHD versus No GVHD. aGVHD acute GVHD, a+cGVHD acute and chronic GVHD, allo-HSCT allogeneic hematopoietic stem cell transplantation, cGVHD chronic GVHD, GVHD graft-versus-host disease.

The mean time to first infection for patients with aGVHD was 10.0 (SD 0.5) months, compared with 15.6 (SD 0.7) months for patients with no GVHD (HR 2.5 [95% CI 1.9, 3.3], *p* < 0.001, for patients who had their first infection recorded after 2 months), and 10.1 (SD 0.1) months for a+cGVHD compared with 16.4 (SD 0.8) months for no GVHD (HR 2.5 [95% CI 1.7, 3.8], *p* < 0.001, for patients who had their first infection recorded after 6 months). The mean time to first infection was not statistically different for patients with cGVHD compared with no GVHD (16.0 [SD 1.3] months versus 16.4 [SD 1.4] months, respectively; HR 0.9 [95% CI 0.8, 1.1], *p* = 0.292). KM curves in Fig. 3 illustrate the time to first severe infection for each GVHD type versus no GVHD.

**Fig. 4:**
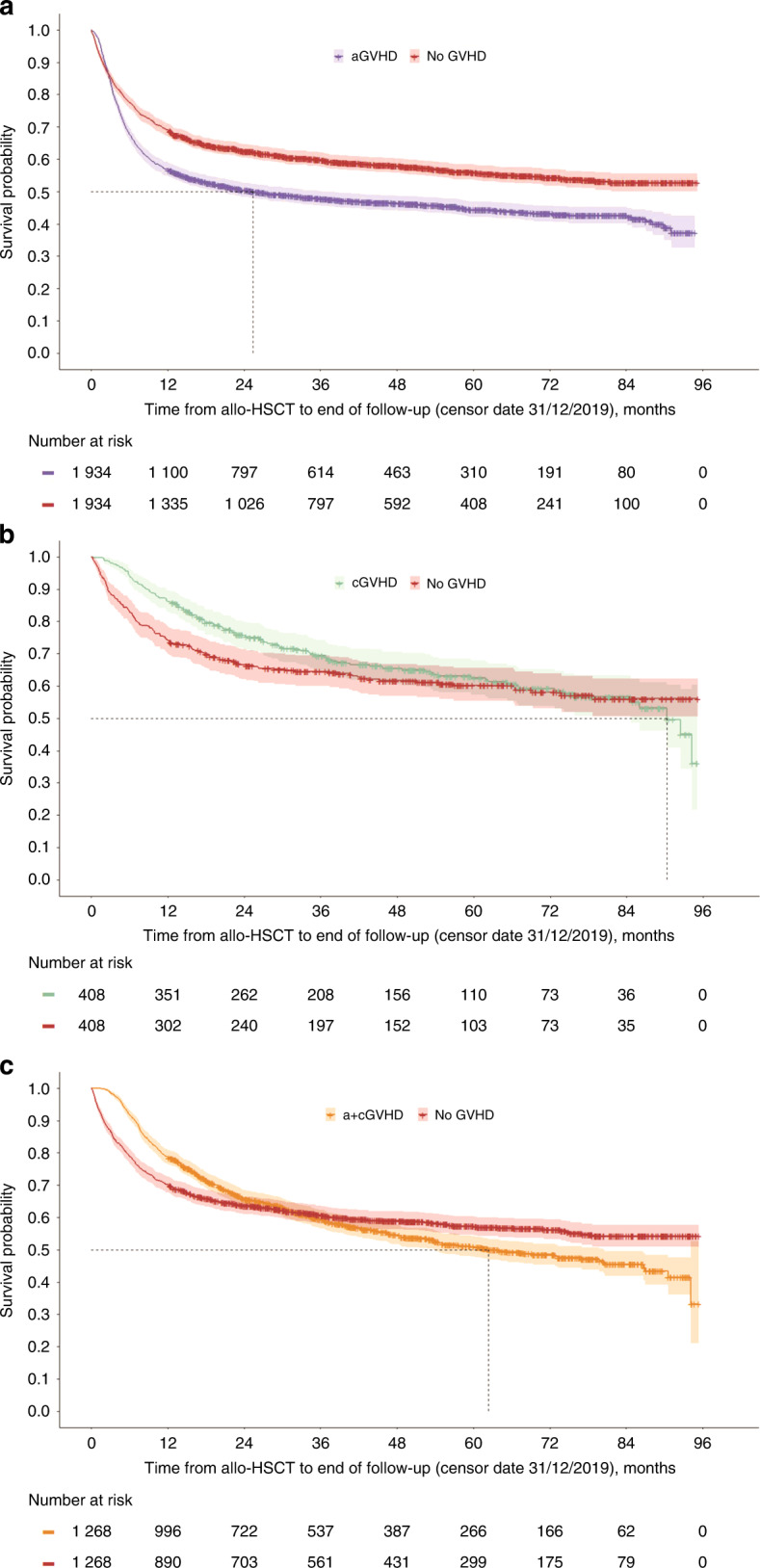
Time to death. **a** aGVHD versus No GVHD. **b** cGVHD versus No GVHD. **c** a + cGVHD versus No GVHD. aGVHD acute GVHD, a+cGVHD acute and chronic GVHD, allo-HSCT allogeneic hematopoietic stem cell transplantation, cGVHD chronic GVHD, GVHD graft-versus-host disease.

Patients with aGVHD had an increased mortality rate (RR 1.6 [95% CI 1.4, 1.7], *p* < 0.05) versus patients with no GVHD; the mortality rate was slightly higher, although not statistically significant, for the a+cGVHD versus no GVHD groups (RR 1.1 [95% CI 1.0, 1.2], *p* > 0.05) and similar between patients with cGVHD and patients with no GVHD (RR 0.9 [95% CI 0.7, 1.1], *p* > 0.05). KM curves in Fig. 4 illustrate the time to death for each GVHD type versus no GVHD.

### Resource and cost implications of GVHD

Patients with aGVHD and a+cGVHD had significantly more overnight hospitalizations per patient-year than patients with no GVHD (mean admission rates aGVHD 4.3 versus 3.3 no GVHD, *p* < 0.001; a+cGVHD 4.2 versus 3.2 no GVHD, *p* < 0.001). Mean overnight hospitalizations per patient-year were similar for patients with cGVHD compared with no GVHD (3.0 versus 3.0, respectively, *p* = 0.044) (Table 2).

**Table 2:** Hospitalization numbers and rates for GVHD and no GVHD subgroups.

	aGVHD (*N* = 1934)	No GVHD (*N* = 1934)	*p* value	cGVHD (*N* = 408)	No GVHD (*N* = 408)	*p* value	a + cGVHD (*N* = 1268)	No GVHD (*N* = 1268)	*p* value
Mean (SD) follow-up time (months)	27.2 (26.9)	33.1 (27.7)	–	41.1 (26.7)	38 (28.7)	–	35.7 (25.5)	35 (28.3)	–
Initial hospitalization for allo-HSCT									
Mean (SD) length of stay, days	44.7 (22.2)	38.0 (18.6)	<0.001	36.5 (15.5)	38.9 (24.2)	0.308	44.2 (27.5)	37.0 (14.8)	<0.001
Subsequent hospitalizations during entire follow-up (overnight stays and day cases)									
Patients with ≥1 subsequent hospitalization, *n*	1724	1696	–	406	370	–	1256	1130	–
Subsequent hospitalizations per patient, mean (SD)	23.7 (26.6)	23.9 (25.0)	0.471	41.9 (33.2)	23.3 (22.6)	<0.001	47.8 (43.5)	24.7 (25.4)	<0.001
Crude rate of hospitalizations per patient-year, mean (SD)	17.5 (17.5)	15.6 (18.4)	<0.001	18.0 (16.1)	14.2 (17.6)	<0.001	21.2 (15.5)	15.6 (18.5)	<0.001
Subsequent hospitalizations during entire follow-up (overnight stays only)									
Patients with ≥1 subsequent overnight hospitalization, *n*	1505	1290	–	365	277	–	1186	881	–
Subsequent hospitalizations per patient, mean (SD)	4.2 (4.3)	4.0 (4.1)	0.183	6.7 (7.0)	4.2 (4.6)	<0.001	8.2 (8.5)	4.0 (4.2)	<0.001
Crude rate of hospitalizations per patient-year, mean (SD)	4.3 (5)	3.3 (3.9)	<0.001	3.0 (3.4)	3.0 (3.9)	0.044	4.2 (4.0)	3.2 (4.0)	<0.001

*aGVHD* acute GVHD, *a+cGVHD* acute and chronic GVHD, *allo-HSCT* allogeneic hematopoietic stem cell transplantation, *cGVHD* chronic GVHD, *GVHD* graft-versus-host disease, *SD* standard deviation.

**Fig. 5:**
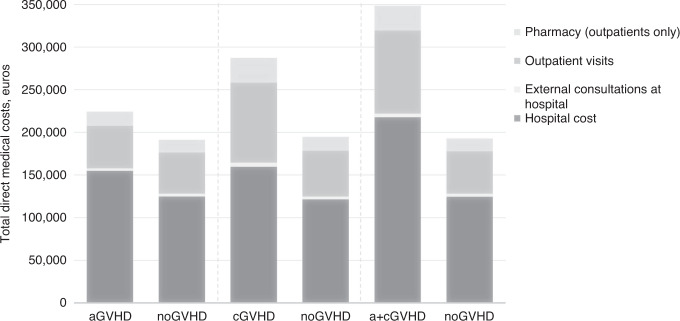
Differences in total direct costs for aGVHD, cGVHD, and a + cGVHD versus no GVHD in the propensity score-matched population, all follow-up. aGVHD acute GVHD, a+cGVHD acute and chronic GVHD, cGVHD chronic GVHD, GVHD graft-versus-host disease. Mean costs per patient are computed among patients with at least one reimbursement for each individual component (the number of patients in the individual component costs can vary). They are presented here as layers; their sum is very close to the total direct cost.

Total direct costs (including hospitalizations, outpatient visits, and pharmacy costs) were 1.2, 1.5, and 1.9 times higher (*p* < 0.001) for patients with aGVHD, cGVHD, and a+cGVHD, respectively, compared with no GVHD. Total indirect costs (including sick leave and transportation) were similar for patients with aGVHD versus patients with no GVHD, 1.3 times higher for patients with cGVHD (*p* = 0.017), and 1.3 times higher for patients with a+cGVHD than those with no GVHD (*p* < 0.001) (Table 3). Mean total cost within 100 days from allo-HSCT was 1.2 times higher for patients with aGVHD (€124,136) compared with no GVHD (€103,173). Hospital cost, including drugs dispensed during hospitalization, was the primary driver of increased HCRU and costs among patients with GVHD (Fig. 5).

**Table 3:** Costs in GVHD and no GVHD subgroups.

	aGVHD (*N* = 1934)	No GVHD (*N* = 1934)	Mean cost ratio *p* value	cGVHD (*N* = 408)	No GVHD (*N* = 408)	Mean cost ratio *p* value	a + cGVHD (*N* = 1268)	No GVHD (*N* = 1268)	Mean cost ratio *p* value
Mean (SD) follow-up time, months	27.2 (26.9)	33.1 (27.7)	–	41.1 (26.7)	38.0 (28.7)	–	35.7 (25.5)	35.0 (28.3)	–
Total direct costs over entire follow-up (includes hospital cost, external consultations at hospital, outpatient visits, and pharmacy), euros									
Mean (SD)	205,305 (13,682)	174,482 (101,468)	1.2 <0.001	272,948 (154,331)	178,004 (105,637)	1.5 <0.001	332,557 (191,575)	175,633 (102,801)	1.9 <0.001
Median (Q1; Q3)	175,636 (135,579; 241,221)	147,485 (114,437; 197,141)	–	234,064 (162,154; 335,449)	147,770 (110,395; 203,770)	–	281,999 (201,878; 409,173)	146,932 (113,470; 200,244)	–
Total indirect and non-medical costs over entire follow-up (includes sick leave and transportation), euros									
Mean (SD)	10,784 (13,374)	10,776 (12,954)	1.0 0.987	15,180 (18,041)	11,992 (14,093)	1.3 0.017	15,316 (18,379)	11,756 (14,091)	1.3 <0.001
Median (Q1; Q3)	5074 (899; 16,994)	5334 (984; 17,074)	–	9777 (1224; 24,493)	6374 (1107; 19,025)	–	8786 (1567; 24,863)	6303 (1142; 18,107)	–
Total all-cause costs over entire follow-up, euros									
Mean (SD)	214,037 (117,205)	183,124 (104,504)	1.2 <0.001	286,490 (160,964)	188,086 (109,831)	1.5 <0.001	346,423 (199,036)	185,118 (106,227)	1.9 <0.001
Median (Q1; Q3)	182,192 (141,563; 251,807)	154,772 (119,844; 208,281)	–	241,563 (172,899; 353,113)	156,061 (117,386; 215,566)	–	292,397 (209,979; 428,280)	155,966 (119,942; 211,463)	–

*aGVHD* acute GVHD, *a+cGVHD* acute and chronic GVHD, *cGVHD* chronic GVHD, *GVHD* graft-versus-host disease, *Q* quartile, *SD* standard deviation.

## Discussion

The clinical and economic impacts of GVHD were demonstrated in this real-world analysis of patients who had an allo-HSCT in France. Compared with patients with no GVHD, those with any type of GVHD were more likely to develop infections and patients with aGVHD had an increased mortality rate. More specifically, patients with aGVHD and a+cGVHD had an increased rate of hospitalizations for severe infections and a shorter time to first infection.

Although numerous changes in transplant practices have improved allo-HSCT results, opportunistic infections remain a serious complication associated with significant morbidity and mortality [15–18]. Beyond financial and economic burden, these potentially life-threatening infections also have important clinical burden and result in poor quality of life [15, 19, 20]. In the current study, 85.3–94.2% patients who presented with GVHD (depending on GVHD type) developed at least one severe infection (versus 81.4–82.2% patients in the matched no GVHD cohorts). In particular, 50.5% patients with a+cGVHD developed four or more severe infections (versus 20.0% patients in the matched no GVHD cohort). These results, in line with previous findings [21], highlight the critical importance of preventing and managing infection for patients receiving allo-HSCT. Indeed, in addition to the prevention, diagnosis, and treatment of the broad range of potential opportunistic infections that may occur after allo-HSCT, decreasing the amount of GVHD with a concomitant improvement of immune responses is key to achieve long-term GVHD and severe infection-free survival [15, 22].

Patients who experienced GVHD, regardless of the GVHD type, had higher HCRU and costs compared with patients who did not experience GVHD. These findings are consistent with previous research [8, 23, 24]. Furthermore, our results were maintained after controlling for key baseline characteristics including age at allo-HSCT, gender, hematological malignancy, type of donor, and type of preparative regimen. These findings are consistent with previous research in the US, which found the clinical and economic burden of GVHD extended for at least a year after transplantation [25].

Patients with GVHD had a longer mean initial length of hospital of stay and a significantly higher number of subsequent hospital stays, including intensive care unit (ICU) visits during these subsequent hospitalizations, compared with patients with no GVHD. During the follow-up period, total direct costs were 1.2, 1.5, and 1.9 times higher for patients with aGVHD, cGVHD, and a+cGVHD, respectively, than for those with no GVHD (*p* < 0.001). These costs were primarily driven by subsequent hospitalizations and drug costs. Patients with aGVHD had a significantly higher number of documented hospitalizations for severe infection as well as a higher rate of mortality than patients with no GVHD. These results are aligned with other studies conducted in Europe [26] and in the US [8–11] which showed increased costs for aGVHD when compared with no GVHD, although the costs differ between these regions. In this study, the mean total costs within 100 days from allo-HSCT were lower than in a similar US study [10], considering an exchange rate of $1.18 = €1. The cost of aGVHD in our study represented 46% of the reported cost for the US study (aGVHD: €124,136 in this study versus US reported cost of $316,458). Similarly, the cost of no GVHD in our study represented 57% of the reported cost for the US study (no GVHD: €103,173 in this study versus US reported cost of $215,229). The difference in costs between the two studies likely reflects country-specific healthcare practice patterns, labor and supply costs.

Compared with patients with no GVHD, patients with cGVHD had a significantly higher number of subsequent hospitalizations, and a higher number of ICU visits during subsequent hospitalizations. Total median indirect and direct costs were significantly higher for patients with cGVHD than no GVHD; these results were also observed for costs per patient-year. Although there was no statistically significant difference between the cGVHD and no GVHD cohorts in the number of patients with severe infection, the proportion of patients with severe infection was numerically higher in the cGVHD cohort. Mortality was similar between patients with cGVHD and those with no GVHD.

Significantly more patients with a+cGVHD had at least one subsequent hospitalization and a higher number of ICU visits during the subsequent hospitalizations, compared with those with no GVHD. Both total median indirect and direct costs as well as costs per patient-year were significantly higher for patients with a+cGVHD compared with those with no GVHD. The number of patients with severe infection was significantly higher for patients with a+cGVHD compared with those with no GVHD, resulting in a higher rate of severe infection. Patients with a+cGVHD had a slightly higher rate of mortality during the study follow-up.

### Limitations

As with all database analyses, this study has limitations. The cohorts were defined using diagnosis codes. Patients may have been misidentified because of coding errors or changes in coding procedures during the course of the study, or some patients with GVHD may not have been identified, such as if the patient died during the index allo-HSCT hospitalization. To offset this, multiple checks were performed to ensure that all patients were adequately captured. The nature of aGVHD versus cGVHD may lead to inherent biases in observed outcomes. While it is difficult to conclude the reason patients with no GVHD, compared with those with cGVHD, had higher mortality, one possible explanation is that patients who survive are more likely to be coded with cGVHD at some point; thus, the mortality outcome is biased on this reasoning. This was not observed for those with aGVHD or a+cGVHD. Relapse-related mortality, as well as relapse-related costs in the no GVHD subgroup, would be interesting to explore further. Finally, the French SNDS database and linked datasets are claims and hospital practice datasets where missing data are possible and difficult to quantify. In particular, GVHD prophylaxis data was not fully captured.

In conclusion, in this nationwide population of French recipients of allo-HSCT, GVHD (in particular, aGVHD and a+cGVHD) was associated with significant clinical and economic burden, including higher rates of infection and mortality as well as increased hospitalizations and direct costs, compared with no GVHD. The results of this study highlight the significant unmet need for effective prophylaxis and treatment options for GVHD, which could reduce or prevent the clinical burden borne by patients experiencing GVHD of all types (in particular severe infections, and thus the need for GVHD treatments that do not further increase the risk of infection), the increased cost of allo-HSCT procedure due to aGVHD, and the potential development of cGVHD, itself leading to further increase in HCRU and costs. Recent advances in allo-HSCT technology, especially in the area of GVHD prevention and treatment, could add to the drug cost; however, it should also reduce the risk of complications and hence the overall clinical and economic burden.

## Supplementary information

Supplementary Table 1

Supplementary Table 2

Supplementary Table 3

## Data Availability

The patient-level data used for this study are not publicly available due to privacy restrictions. The aggregated data generated during the current study are available from the corresponding author on reasonable request.
