# Supplementary material for: Clinical and economic burden associated with graft-versus-host disease following allogeneic hematopoietic cell transplantation in France
Source: Bone Marrow Transplant. 2023 Feb 10;58(5):514–25. doi: 10.1038/s41409-023-01930-8 (PMC10162942; doi:10.1038/s41409-023-01930-8)
Supplement: Supplementary file 1 — Supplementary Table 1 [file 41409_2023_1930_MOESM1_ESM.docx]

**Supplementary Table 1. Diagnosis Codes**

**a. Identification of allo-HSCT Procedure**

|  | **Label** | **Code** |
| --- | --- | --- |
| **CCAM** | Intravenous injection of an allograft cell therapy product | [FELF009](https://www.aideaucodage.fr/ccam-felf009) |
| **DRG** | Hematopoietic stem cell allografts, level 1 | 27Z021 |
|  | Hematopoietic stem cell allografts, level 2 | 27Z022 |
|  | Hematopoietic stem cell allografts, level 3 | 27Z023 |
|  | Hematopoietic stem cell allografts, level 4 | 27Z024 |

allo-HSCT, allogeneic hematopoietic stem cell transplantation; CCAM, Classification commune des actes médicaux; DRG, Diagnosis-related group.

**b. Identification of GVHD**

|  | **Label** | **ICD-10 code** |
| --- | --- | --- |
| **ICD-10** | Acute graft versus host disease | T86.01 |
|  | Chronic graft versus host disease | T86.02 |

aGVHD: Patients with at least a relevant code for aGVHD (ICD-10: T86.01) during follow-up period, without any cGVHD code.

cGVHD: Patients with at least a relevant code for cGVHD (ICD-10: T86.02) during follow-up period, without any aGVHD code.

a+cGVHD: Patients with at least one of each relevant codes for aGVHD (ICD-10: T86.01) and cGVHD (ICD-10: T86.02) during follow-up.

aGVHD, acute GVHD; a+cGVHD, acute and chronic GVHD; cGVHD, chronic GVHD; GVHD, graft-versus-host disease; ICD-10, International Classification of Diseases, 10^th^ revision.

**c. Identification of Severe Infections**

| **Infection** | **ICD-10 code** |
| --- | --- |
| **Viral** |  |
| Varicella zoster virus | B01 |
| Cytomegalovirus | B25, B271 |
| Epstein-Barr virus | A858, B348, B270, D823 |
| Adenovirus | A082, A851, A871, B178, B300, B301, B340, B970, J120 |
| Human herpesvirus/Herpes simplex virus | B00, A60, H191 |
| Human papillomavirus | B977 |
| Parainfluenza virus | J122, J204 |
| Respiratory syncytial virus | J121, J210, B974, J205 |
| Meningitis | G00, G03, A390, A398, A399, B003,A321, G01, A170, G02, B010, A87, B384, B021, B261, B375 |
| Viral encephalitis | A86, A85, A83, A84 |
| Lower respiratory tract infections | J22, J440 |
| Herpes zoster virus | B02 |
| Other viral infections | B08, B09, B271, B278, B279, B33, B34 |
| **Bacterial** |  |
| Bacterial infections | J15, J170, A48, A49, B95, B96 |
| Severe sepsis | A41, A40, A391, A392, A393, A394, A427 |
| Brain abscess | G060, G09, T798, T799 |
| Clostridium difficile | A047 |
| **Fungal** |  |
| Aspergillosis | B44 |
| Candidiasis | B37 |
| Other mycoses | B48 |
| Unspecified mycoses | B49 |
| Zygomycosis | B46 |
| Pneumocystosis | B59, J172 |
| **Other** |  |
| Toxoplasmosis | B58 |
| Strongyloides infection | B78 |
| Sepsis, unspecified infection | R650, R651, R572 |

ICD-10, International Classification of Diseases, 10^th^ revision

**d. Identification of Relapse – Cancer treatments**

| **Cancer treatment** | ATC code |
| --- | --- |
| Ivosidenib | L01XX62 |
| Enasidenib | L01XX59 |
| Gilteritinib | L01XE54 |
| Cytarabine | L01BC01, L01XY01 |
| Azacitidine | L01BC07 |
| Cladribine | L01BB04, L04AA40 |
| Fludarabine | L01BB05 |
| Idarubicin | L01DB06 |
| Doxorubicin | L01DB01 |
| Daunorubicin | L01DB02, L01XY01 |
| Mitoxantrone | L01DB07 |
| Etoposide | L01CB01 |
| Gemtuzumab ozogamicin | L01XC05 |

ATC, Anatomical Therapeutic Chemical
