# Supplementary material for: Clinical and economic burden associated with graft-versus-host disease following allogeneic hematopoietic cell transplantation in France
Source: Bone Marrow Transplant. 2023 Feb 10;58(5):514–25. doi: 10.1038/s41409-023-01930-8 (PMC10162942; doi:10.1038/s41409-023-01930-8)
Supplement: Supplementary file 2 — Supplementary Table 2 [file 41409_2023_1930_MOESM2_ESM.docx]

**Supplementary Table 2. Patient Characteristics Before Propensity Score Matching**

|  | **Allo-HSCT**  **(N=6 385)** | | **aGVHD**  **(N=2 002)** | | **cGVHD**  **(N=411)** | | **a+cGVHD**  **(N=1 304)** | | **No GVHD**  **(N=2 668)** | | **p-value^a^** |
| --- | --- | --- | --- | --- | --- | --- | --- | --- | --- | --- | --- |
| Hematological condition for allo-HSCT^b^ (n, %) |  |  | |  | |  | |  | |  | |
| Myeloid leukemia | 3 053 (47.8) | 897 (44.8) | | 203 (49.4) | | 618 (47.4) | | 1 335 (50.0) | | 0.005 | |
| Acute myeloblastic leukemia | 2 631 (41.2) | 760 (38.0) | | 179 (43.6) | | 517 (39.6) | | 1 175 (44.0) | | 0.000 | |
| Lymphoid leukemia | 1 137 (17.8) | 406 (20.3) | | 68 (16.6) | | 229 (17.6) | | 434 (16.3) | | 0.004 | |
| Myelodysplastic syndromes | 878 (13.8) | 304 (15.2) | | 56 (13.6) | | 185 (14.2) | | 333 (12.5) | | 0.063 | |
| Acute lymphoblastic leukemia | 823 (12.9) | 307 (15.3) | | 41 (10.0) | | 154 (11.8) | | 321 (12.0) | | 0.001 | |
| Non-Hodgkin lymphoma | 368 (5.8) | 108 (5.4) | | 23 (5.6) | | 68 (5.2) | | 169 (6.3) | | 0.416 | |
| Multiple myeloma and plasma cell neoplasms | 351 (5.5) | 98 (4.9) | | 24 (5.8) | | 72 (5.5) | | 157 (5.9) | | 0.520 | |
| Hodgkin lymphoma | 288 (4.5) | 90 (4.5) | | 18 (4.4) | | 65 (5.0) | | 115 (4.3) | | 0.814 | |
| Monocytic leukemia | 193 (3.0) | 63 (3.2) | | 11 (2.7) | | 44 (3.4) | | 75 (2.8) | | 0.746 | |
|  |  |  | |  | |  | |  | |  | |
| Chronic lymphocytic leukemia | 191 (3.0) | 57 (2.8) | | 16 (3.9) | | 49 (3.8) | | 69 (2.6) | | 0.142 | |
| Acute myelomonocytic leukemia | 123 (1.9) | 34 (1.7) | | 13 (3.2) | | 28 (2.2) | | 48 (1.8) | | 0.217 | |
| Chronic myeloid leukemia | 113 (1.8) | 31 (1.6) | | Freq <5 (0) | | 33 (2.5) | | 46 (1.7) | | 0.058 | |
| Other and unspecified malignant neoplasms of the lymphatic, hematopoietic, and related tissues | 16 (0.2) | Freq <5 (0) | | Freq <5 (0) | | 6 (0.5) | | 8 (0.3) | | 0.145 | |
| Not specified^c^ | 101 (1.6) | 34 (1.7) | | 8 (2.0) | | 17 (1.3) | | 42 (1.6) | | 0.759 | |
| Year of allo-HSCT (n, %) |  |  | |  | |  | |  | |  | |
| 2012 | 699 (11.0) | 197 (9.8) | | 58 (14.1) | | 151 (11.6) | | 293 (11.0) | | 0.353 | |
| 2013 | 879 (13.8) | 266 (13.3) | | 62 (15.1) | | 185 (14.2) | | 366 (13.7) | |  |  |
| 2014 | 940 (14.7) | 288 (14.4) | | 54 (13.1) | | 203 (15.6) | | 395 (14.8) | |  |  |
| 2015 | 960 (15.0) | 307 (15.3) | | 62 (15.1) | | 191 (14.7) | | 400 (15.0) | |  |  |
| 2016 | 945 (14.8) | 310 (15.5) | | 60 (14.6) | | 202 (15.5) | | 373 (14.0) | |  |  |
| 2017 | 943 (14.8) | 296 (14.8) | | 52 (12.7) | | 196 (15.0) | | 399 (15.0) | |  |  |
| 2018 | 1 019 (16.0) | 338 (16.9) | | 63 (15.3) | | 176 (13.5) | | 442 (16.6) | |  |  |
| Age at allo-HSCT^b^ (n, %) |  |  | |  | |  | |  | |  | |
| Mean (SD) | 51.1 (13.7) | 51.2 (13.8) | | 51.4 (13.1) | | 50.7 (13.2) | | 51.1 (13.9) | | 0.427 | |
| Median (Q1, Q3) | 54.0 (42.0, 62.0) | 54.5 (42.0 62.0) | | 54.0 (42.0, 62.0) | | 53.0 (42.0, 61.0) | | 54.0 (42.0, 62.0) | |  |  |
| Range (min, max) | (18.0, 8.0) | (18.0, 77.0) | | (18.0, 75.0) | | (18.0, 76.0) | | (18.0, 78.0) | |  |  |
| Age group |  |  | |  | |  | |  | |  | |
| 18 to <25 years | 344 (5.4) | 119 (5.9) | | 13 (3.1) | | 62 (4.8) | | 150 (5.6) | | 0.031 | |
| 25 to <45 years | 1 487 (23.3) | 440 (22.0) | | 114 (27.7) | | 318 (24.4) | | 615 (23.0) | |  |  |
| 45 to <65 years | 3 509 (55.0) | 1 103 (55.1) | | 212 (51.6) | | 739 (56.7) | | 1 455 (54.5) | |  |  |
| >65 years | 1 045 (16.4) | 340 (17.0) | | 72 (17.5) | | 185 (14.2) | | 448 (16.8) | |  |  |
| Age at first GVHD (n, %) |  |  | |  | |  | |  | |  | |
| Mean (SD) |  | 51.3 (13.8) | | 52.2 (13.1) | | 50.9 (13.2) | |  | |  | |
| Median (Q1, Q3) |  | 55.0 (42.0, 62.0) | | 55.0 (43.0, 63.0) | | 53.0 (42.0, 62.0) | |  | |  | |
| Range (min, max) |  | (18.0, 77.0) | | (18.0, 75.0) | | (18.0, 76.0) | |  | |  | |
| Age group |  |  | |  | |  | |  | |  | |
| 18 to <25 years |  | 118 (5.9) | | 12 (2.9) | | 62 (4.8) | |  | |  | |
| 25 to <45 years |  | 436 (21.8) | | 106 (25.8) | | 312 (23.9) | |  | |  | |
| 45 to <65 years |  | 1 105 (55.2) | | 206 (50.1) | | 735 (56.4) | |  | |  | |
| >65 years |  | 343 (17.1) | | 87 (21.2) | | 195 (15.0) | |  | |  | |
| Gender (n, %) |  |  | |  | |  | |  | |  | |
| Male | 3 695 (57.9) | 1 163 (58.1) | | 218 (53.0) | | 754 (57.8) | | 1 560 (58.5) | | 0.225 | |
| Female | 2 690 (42.1) | 839 (41.9) | | 193 (47.0) | | 550 (42.2) | | 1 108 (41.5) | |  |  |
| Donor typing (n, %) |  |  | |  | |  | |  | |  | |
| Related | 1 636 (25.6) | 471 (23.5) | | 115 (28.0) | | 302 (23.2) | | 748 (28.0) | |  | |
| Unrelated | 2 232 (35.0) | 758 (37.9) | | 140 (34.1) | | 459 (35.2) | | 875 (32.8) | |  | |
| NA | 2 574 (40.3) | 790 (39.5) | | 159 (38.7) | | 552 (42.3) | | 1 073 (40.2) | |  | |
| Preparative regimens^d^ (n, %) |  |  | |  | |  | |  | |  | |
| Yes | 5 811 (91.0) | 1 825 (91.2) | | 382 (92.9) | | 1 169 (89.6) | | 2 435 (91.3) | | 0.164 | |
| Busulfan | 4 603 (72.1) | 1 412 (70.5) | | 311 (75.7) | | 936 (71.8) | | 1 944 (72.9) | | 0.117 | |
| Total body irradiation | 1 197 (18.8) | 412 (20.6) | | 71 (17.3) | | 232 (17.8) | | 482 (18.1) | | 0.086 | |
| Clofarabine | 287 (4.5) | 85 (4.2) | | 12 (2.9) | | 37 (2.8) | | 153 (5.7) | | <0.001 | |
| Carmustine | 19 (0.3) | 5 (0.2) | | Freq <5 (0) | | 13 (1.0) | | Freq <5 (0) | | <0.001 | |
| Alemtuzumab | Freq <5 (0) | Freq <5 (0) | | Freq <5 (0) | | Freq <5 (0) | | Freq <5 (0) | | 0.426 | |
| Antithymocyte immunoglobulin | Freq <5 (0) | Freq <5 (0) | | Freq <5 (0) | | Freq <5 (0) | | Freq <5 (0) | | NA | |
| Cyclophosphamide | Freq <5 (0) | Freq <5 (0) | | Freq <5 (0) | | Freq <5 (0) | | Freq <5 (0) | | NA | |
| Cytarabine | Freq <5 (0) | Freq <5 (0) | | Freq <5 (0) | | Freq <5 (0) | | Freq <5 (0) | | 0.002 | |
| Etoposide | Freq <5 (0) | Freq <5 (0) | | Freq <5 (0) | | Freq <5 (0) | | Freq <5 (0) | | NA | |
| Fludarabine | Freq <5 (0) | Freq <5 (0) | | Freq <5 (0) | | Freq <5 (0) | | Freq <5 (0) | | NA | |
| Melphalan | Freq <5 (0) | Freq <5 (0) | | Freq <5 (0) | | Freq <5 (0) | | Freq <5 (0) | | NA | |
| Thiotepa | Freq <5 (0) | Freq <5 (0) | | Freq <5 (0) | | Freq <5 (0) | | Freq <5 (0) | | NA | |
| Treosulfan | Freq <5 (0) | Freq <5 (0) | | Freq <5 (0) | | Freq <5 (0) | | Freq <5 (0) | | NA | |
| Not captured | 574 (9.0) | 177 (8.8) | | 29 (7.1) | | 135 (10.4) | | 233 (8.7) | | NA | |
| GVHD prophylaxis^e^ (n, %) |  |  | |  | |  | |  | |  | |
| Yes |  | 658 (32.9) | | 377 (91.7) | | 547 (42.0) | | 2 164 (81.1) | |  | |
| Ciclosporin |  | 612 (30.6) | | 352 (85.6) | | 507 (38.9) | | 2 064 (77.4) | |  | |
| Mycophenolate mofetil |  | 335 (16.7) | | 143 (34.8) | | 247 (18.9) | | 845 (31.7) | |  | |
| Tacrolimus |  | 40 (2.0) | | 53 (12.9) | | 43 (3.3) | | 123 (4.6) | |  | |
| Sirolimus |  | 5 (0.3) | | 10 (2.4) | | 5 (0.4) | | 16 (0.7) | |  | |
| Antithymocyte immunoglobulin |  | Freq <5 (0) | | Freq <5 (0) | | Freq <5 (0) | | Freq <5 (0) | |  | |
| Methotrexate |  | Freq <5 (0) | | 5 (1.2) | | Freq <5 (0) | | Freq <5 (0) | |  | |
| Not captured |  | 1 344 (67.1) | | 34 (8.3) | | 757 (58.0) | | 504 (18.9) | |  | |
| Comorbidities^f^ (n, %) |  |  | |  | |  | |  | |  | |
| Any tumor (including lymphoma and leukemia except for malignant neoplasm of skin) | 5 833 (91.4) | 1 804 (90.1) | | 372 (90.5) | | 1 182 (90.6) | | 2 475 (92.8) | | 0.008 | |
| Diabetes | 668 (10.5) | 218 (10.9) | | 44 (10.7) | | 166 (12.7) | | 240 (9) | | 0.003 | |
| Chronic pulmonary disease | 535 (8.4) | 173 (8.6) | | 34 (8.3) | | 108 (8.3) | | 220 (8.3) | | 0.967 | |
| Congestive heart failure | 517 (8.1) | 154 (7.7) | | 24 (5.8) | | 96 (7.4) | | 243 (9.1) | | 0.049 | |
| Moderate to severe liver disease | 278 (4.4) | 96 (4.8) | | 13 (3.2) | | 49 (3.8) | | 120 (4.5) | | 0.310 | |
| Cerebrovascular disease | 239 (3.7) | 84 (4.2) | | 14 (3.4) | | 22 (1.7) | | 119 (4.5) | | <0.001 | |
| Mild liver disease | 207 (3.2) | 67 (3.4) | | 18 (4.4) | | 44 (3.4) | | 78 (2.9) | | 0.440 | |
| Metastatic solid tumor | 202 (3.2) | 58 (2.9) | | 15 (3.6) | | 45 (3.4) | | 84 (3.2) | | 0.769 | |
| Moderate or severe renal disease | 180 (2.8) | 62 (3.1) | | 10 (2.4) | | 31 (2.4) | | 77 (2.9) | | 0.624 | |
| Myocardial infarction | 111 (1.7) | 31 (1.6) | | 6 (1.5) | | 17 (1.3) | | 57 (2.1) | | 0.210 | |
| Hemiplegia | 100 (1.6) | 31 (1.6) | | Freq <5 (0) | | 14 (1.1) | | 52 (2.0) | | 0.091 | |
| Peripheral vascular disease | 109 (1.7) | 34 (1.7) | | Freq <5 (0) | | 22 (1.7) | | 49 (1.8) | | 0.662 | |
| Ulcer disease | 68 (1.1) | 28 (1.4) | | 6 (1.5) | | 12 (0.9) | | 22 (0.8) | | 0.217 | |
| Connective tissue disease | 46 (0.7) | 11 (0.6) | | 6 (1.5) | | 14 (1.1) | | 15 (0.6) | | 0.067 | |
| Diabetes with end-organ damage | 11 (0.2) | Freq <5 (0) | | Freq <5 (0) | | Freq <5 (0) | | Freq <5 (0) | | 0.917 | |
| HIV/AIDS | 9 (0.1) | 5 (0.3) | | Freq <5 (0) | | Freq <5 (0) | | Freq <5 (0) | | 0.362 | |
| Dementia | 5 (0.1) | Freq <5 (0) | | Freq <5 (0) | | Freq <5 (0) | | Freq <5 (0) | | 0.931 | |
| CCI (12 months prior to index) |  |  | |  | |  | |  | |  | |
| Mean (SD) | 2.6 (1.7) | 2.6 (1.7) | | 2.6 (1.7) | | 2.6 (1.7) | | 2.7 (1.7) | | 0.082 | |
| Median (Q1, Q3) | 2.0 (2.0, 3.0) | 2.0 (2.0, 3.0) | | 2.0 (2.0, 3.0) | | 2.0 (2.0, 3.0) | | 2.0 (2.0, 3.0) | |  |  |
| Range (min, max) | (0.0, 15.0) | (0.0, 12.0) | | (0.0, 11.0) | | (0.0, 11.0) | | (0.0, 15.0) | |  |  |
| CCI category (n, %) |  |  | |  | |  | |  | |  | |
| 0 | 377 (5.9) | 129 (6.4) | | 29 (7.1) | | 83 (6.4) | | 136 (5.1) | | 0.074 | |
| 1 | 110 (1.7) | 46 (2.3) | | 9 (2.2) | | 22 (1.7) | | 33 (1.2) | |  |  |
| 2 | 3 649 (57.2) | 1 114 (55.6) | | 236 (57.4) | | 769 (59.0) | | 1 530 (57.4) | |  |  |
| 3 | 1 250 (19.6) | 385 (19.2) | | 79 (19.2) | | 237 (18.2) | | 549 (20.6) | |  |  |
| ≥4 | 999 (15.7) | 328 (16.4) | | 58 (14.1) | | 193 (14.8) | | 420 (15.7) | |  |  |
| Follow-up from first allo-HSCT (days) |  |  | |  | |  | |  | |  | |
| Mean (SD) | 977.2 (830.4) | 824.4 (815.2) | | 1 245.7 (812.6) | | 1 090.3 (782.1) | | 995.2 (846.4) | |  | |
| Median (Q1; Q3) | 740.0 (225.0; 1 613.0) | 487.5 (135.0; 1 398.0) | | 1 121.0 (532.0; 1 934.0) | | 917.0 (421.0; 1 644.0) | | 769.5 (215.5; 1 679.0) | |  | |
| Range (min, max) | (1.0; 2 904.0) | (1.0; 2 885.0) | | (10.0; 2 896.0) | | (27.0; 2 904.0) | | (1.0; 2 904.0) | |  | |
| Follow-up from first allo-HSCT (months) |  |  | |  | |  | |  | |  | |
| Mean (SD) | 32.1 (27.3) | 27.1 (26.8) | | 40.9 (26.7) | | 35.8 (25.7) | | 32.7 (27.8) | |  | |
| Median (Q1, Q3) | 24.3 (7.4, 53.0) | 16.0 (4.4, 45.9) | | 36.8 (17.5, 63.5) | | 30.1 (13.8, 54.0) | | 25.3 (7.1, 55.2) | |  | |
| Range (min, max) | (0.0, 95.4) | (0.0, 94.8) | | (0.3, 95.1) | | (0.9, 95.4) | | (0.0, 95.4) | |  | |

aGVHD, acute GVHD; a+cGVHD, acute and chronic GVHD; AIDS, acquired immunodeficiency syndrome; allo-HSCT, allogeneic hematopoietic stem cell transplantation; ANOVA, analysis of variance; CCI, Charlson Comorbidity Index; cGVHD, chronic GVHD; GVHD, graft-versus-host disease; HIV, human immunodeficiency virus; HSCT, hematopoietic stem cell transplantation; max, maximum; min, minimum; NA, not applicable due to low sample size; Q, quartile; SD, standard deviation.

^a^For continuous variables, if the normality test showed that the normal distribution assumption was not true, a Kruskal-Wallis test was used. If normality and homogeneity of variance were satisfied, then 1-way ANOVA test was used. Chi-square test was used for categorical variables. If 25% of the cells had expected counts <5, then a Fisher Exact test was applied.

^b^Most recent hematological condition before the admission date of allo-HSCT (including the date of allo-HSCT); conditions were exclusive.

^c^Any patient with different malignancy conditions on the same day was regarded as not specified.

^d^Preparative regimens administered after admission prior to allo-HSCT procedure (including the day of allo-HSCT); regimens were not exclusive.

^e^GVHD prophylaxis was administrated before the first documented GVHD and after the first allo-HSCT for patients with any type of GVHD; GVHD prophylaxis for the no GVHD group focused on prophylaxis within 100 days after the first allo-HSCT.

^f^At least one comorbidity within 1 year before the first allo-HSCT; these comorbidities were not mutually exclusive.
