# Supplementary material for: Clinical and economic burden associated with graft-versus-host disease following allogeneic hematopoietic cell transplantation in France
Source: Bone Marrow Transplant. 2023 Feb 10;58(5):514–25. doi: 10.1038/s41409-023-01930-8 (PMC10162942; doi:10.1038/s41409-023-01930-8)
Supplement: Supplementary file 3 — Supplementary Table 3 [file 41409_2023_1930_MOESM3_ESM.docx]

**Supplementary Table 3. Patients with a Documented Hospitalization Code for Severe Infection, Overall and by Type of Infection**

|  | **aGVHD**  **(N=1 934)** | **No GVHD**  **(N=1 934)** | **Odds ratio (95% CI)** | **p-value** | **cGVHD**  **(N=408)** | **No GVHD**  **(N=408)** | **Odds ratio (95% CI)** | **p-value** | **a+c GVHD**  **(N=1 268)** | **No GVHD**  **(N=1 268)** | **Odds ratio (95% CI)** | **p-value** |
| --- | --- | --- | --- | --- | --- | --- | --- | --- | --- | --- | --- | --- |
| **Number of patients with a documented hospitalization code for severe infection (n, %)** | **1 720 (88.9)** | **1 589 (82.2)** | **1.7**  **(1.4, 2.1)** | **<0.001** | **348 (85.3)** | **334 (81.9)** | 1.3  (0.9, 1.9) | 0.179 | **1 195 (94.2)** | **1 032 (81.4)** | **4**  **(3, 5.4)** | **<0.001** |
| **Patients with viral infection (n, %)** | 916 (47.4) | 532 (27.5) | **2.3**  **(2, 2.6)** | **<0.001** | 183 (44.9) | 130 (31.9) | **1.9**  **(1.4, 2.5)** | **0.001** | 750 (59.1) | 362 (28.5) | **3.6**  **(3, 4.3)** | **<0.001** |
| Varicella zoster virus | 15 (0.8) | 9 (0.5) | 1.7  (0.7, 3.8) | 0.226 | Freq < 5 | Freq < 5 | 1.5  (0.3, 9) | 0.657 | 10 (0.8) | 8 (0.6) | 1.3  (0.5, 3.2) | 0.638 |
| CMV infection | 554 (28.6) | 250 (12.9) | **2.7**  **(2.3, 3.2)** | **<0.001** | 96 (23.5) | 56 (13.7) | **1.9**  **(1.3, 2.8)** | **0.001** | 456 (36) | 158 (12.5) | **4**  **(3.2, 5)** | **<0.001** |
| EBV infection | 134 (6.9) | 67 (3.5) | **2.1**  **(1.5, 2.8)** | **<0.001** | 20 (4.9) | 18 (4.4) | 1.1  (0.6, 2.2) | 0.739 | 104 (8.2) | 41 (3.2) | **2.8**  **(1.9, 4)** | **<0.001** |
| Adenovirus | 59 (3.1) | 25 (1.3) | **2.4**  **(1.5, 3.8)** | **<0.001** | 10 (2.5) | 5 (1.2) | 2  (0.7, 5.9) | 0.206 | 58 (4.6) | 10 (0.8) | **5.8**  **(3, 11)** | **<0.001** |
| HHV/HSV infection | 192 (9.9) | 121 (6.3) | **1.7**  **(1.3, 2.1)** | **<0.001** | 37 (9.1) | 28 (6.9) | 1.3  (0.8, 2.2) | 0.251 | 190 (15) | 83 (6.5) | **2.5**  **(1.9, 3.3)** | **<0.001** |
| Parainfluenza virus | 7 (0.4) | 6 (0.3) | 1.2  (0.4, 3.5) | 0.782 | 6 (1.5) | Freq < 5 | 6  (0.7, 49.8) | 0.097 | 24 (1.9) | 5 (0.4) | **4.8**  **(1.8, 13)** | **0.001** |
| Respiratory syncytial virus | 43 (2.2) | 30 (1.6) | 1.4  (0.9, 2.3) | 0.125 | 17 (4.2) | 6 (1.5) | **3.7**  **(1.2, 11.3)** | **0.019** | 73 (5.8) | 20 (1.6) | **4.3**  **(2.5, 7.4)** | **<0.001** |
| Meningitis | 44 (2.3) | 30 (1.6) | 1.5  (0.9, 2.3) | 0.106 | 7 (1.7) | 8 (2) | 0.9  (0.3, 2.4) | 0.796 | 30 (2.4) | 26 (2.1) | 1.2  (0.7, 2) | 0.593 |
| Viral encephalitis | 15 (0.8) | 4 (0.2) | **3.7**  **(1.2, 11)** | **0.019** | Freq < 5 | Freq < 5 | 3  (0.3, 28.8) | 0.341 | 10 (0.8) | Freq < 5 | 3.3  (0.9, 12) | 0.067 |
| Lower tract respiratory infections | 17 (0.9) | 12 (0.6) | 1.5  (0.7, 3.1) | 0.339 | 16 (3.9) | Freq < 5 | **5.3**  **(1.6, 18.3)** | **0.008** | 62 (4.9) | 7 (0.6) | **8.9**  **(4.1, 19)** | **<0.001** |
| Herpes zoster virus | 48 (2.5) | 38 (2) | 1.3  (0.8, 2) | 0.276 | 14 (3.4) | 11 (2.7) | 1.3  (0.6, 2.8) | 0.549 | 48 (3.8) | 27 (2.1) | **1.8**  **(1.1, 3)** | **0.014** |
| Other viral infections | 211 (10.9) | 114 (5.9) | **2**  **(1.5, 2.5)** | **<0.001** | 40 (9.8) | 33 (8.1) | **1.2**  **(0.8, 2.1)** | **0.379** | 208 (16.4) | 75 (5.9) | **3.1**  **(2.4, 4.2)** | **<0.001** |
| **Patients with bacterial infection (n, %)** | 1 504 (77.8) | 1 427 (73.8) | **1.2**  **(1.1, 1.4)** | **0.004** | 322 (78.9) | 292 (71.6) | **1.5**  **(1.1, 2.1)** | **0.014** | 1 101 (86.8) | 920 (72.6) | **2.5**  **(2, 3.1)** | **<0.001** |
| Bacterial infections | 1 033 (53.4) | 876 (45.3) | **1.4**  **(1.2, 1.6)** | **<0.001** | 249 (61) | 177 (43.4) | **2.1**  **(1.6, 2.8)** | **0.001** | 875 (69) | 563 (44.4) | **2.8**  **(2.4, 3.4)** | **<0.001** |
| Severe sepsis | 988 (51.1) | 898 (46.4) | **1.2**  **(1.1, 1.4)** | **0.004** | 214 (52.5) | 176 (43.1) | **1.4**  **(1.1, 1.9)** | **0.009** | 773 (61) | 584 (46.1) | **1.8**  **(1.6, 2.2)** | **<0.001** |
| Brain abscess | 10 (0.5) | 7 (0.4) | 1.4  (0.5, 3.8) | 0.469 | Freq < 5 | Freq < 5 | NA | NA | 16 (1.3) | Freq < 5 | **5.3**  **(1.6, 18)** | **0.008** |
| Clostridium difficile | 216 (11.2) | 162 (8.4) | **1.4**  **(1.1, 1.7)** | **0.004** | 28 (6.9) | 33 (8.1) | 0.8  (0.5, 1.4) | 0.493 | 208 (16.4) | 108 (8.5) | **2.1**  **(1.6, 2.6)** | **<0.001** |
| Sepsis, unspecified infection | 770 (39.8) | 719 (37.2) | 1.1  (1, 1.3) | 0.093 | 154 (37.7) | 138 (33.8) | 1.2  (0.9, 1.6) | 0.220 | 620 (48.9) | 467 (36.8) | **1.6**  **(1.4, 1.9)** | **<0.001** |
| **Patients with fungal infection (n, %)** | 613 (31.7) | 483 (25) | **1.4**  **(1.2, 1.6)** | **<0.001** | 134 (32.8) | 101 (24.8) | **1.4**  **(1.1, 1.9)** | **0.015** | 589 (46.5) | 324 (25.6) | **2.5**  **(2.1, 2.9)** | **<0.001** |
| Aspergillosis | 287 (14.8) | 203 (10.5) | **1.5**  **(1.2, 1.8)** | **<0.001** | 59 (14.5) | 44 (10.8) | 1.4  (0.9, 2.1) | 0.125 | 300 (23.7) | 142 (11.2) | **2.5**  **(2, 3.2)** | **<0.001** |
| Candidiasis | 335 (17.3) | 251 (13) | **1.4**  **(1.2, 1.7)** | **<0.001** | 77 (18.9) | 53 (13) | **1.6**  **(1.1, 2.3)** | **0.023** | 304 (24) | 154 (12.1) | **2.2**  **(1.8, 2.8)** | **<0.001** |
| Mycosis unspecified, (Other mycoses) | 36 (1.9) | 31 (1.6) | 1.2  (0.7, 1.9) | 0.536 | 11 (2.7) | Freq < 5 | 2.8  (0.9, 8.6) | 0.083 | 41 (3.2) | 22 (1.7) | **2**  **(1.2, 3.5)** | **0.014** |
| Mucormycosis, unspecified | 3 (0.2) | 4 (0.2) | 0.8  (0.2, 3.4) | 0.706 | Freq < 5 | Freq < 5 | NA | NA | 11 (0.9) | Freq < 5 | **3.7**  **(1, 13)** | **0.046** |
| Zygomycosis/Mycoses from opportunistic-pathogenic fungi | 20 (1) | 16 (0.8) | 1.3  (0.6, 2.4) | 0.506 | Freq < 5 | Freq < 5 | 1.5  (0.3, 9) | 0.657 | 25 (2) | 9 (0.7) | **3**  **(1.3, 6.7)** | **0.007** |
| Pneumocystis | 124 (6.4) | 110 (5.7) | 1.1  (0.9, 1.5) | 0.346 | 43 (10.5) | 24 (5.9) | **1.9**  **(1.1, 3.3)** | **0.015** | 163 (12.9) | 87 (6.9) | **2**  **(1.5, 2.6)** | **<0.001** |
| **Patients with other infection (n, %) -** Toxoplasmosis | 51 (2.6) | 24 (1.2) | **2.2**  **(1.3, 3.7)** | **0.002** | 6 (1.5) | 6 (1.5) | 1  (0.3, 3.1) | 1.000 | 30 (2.4) | 11 (0.9) | **2.7**  **(1.4, 5.4)** | **0.004** |

aGVHD, acute GVHD; a+cGVHD, acute and chronic GVHD; cGVHD, chronic GVHD; CI, confidence interval; CMV, cytomegalovirus; EBV, Epstein-Barr virus; GVHD, graft-versus-host disease; HHV/HSV human herpes virus/herpes simplex virus; NA, not applicable due to low sample size.

Note: Odds ratios and p-values highlighted in **bold** are statistically significant.
